# Human-Induced Landscape Changes Homogenize Atlantic Forest Bird Assemblages through Nested Species Loss

**DOI:** 10.1371/journal.pone.0147058

**Published:** 2016-02-03

**Authors:** Marcelo Alejandro Villegas Vallejos, André Andrian Padial, Jean Ricardo Simões Vitule

**Affiliations:** 1 Hori Consultoria Ambiental, Curitiba, Paraná, Brazil; 2 Departamento de Botânica, Universidade Federal do Paraná, Curitiba, Paraná, Brazil; 3 Programa de Pós-Graduação em Ecologia e Conservação, Setor de Ciências Biológicas, Universidade Federal do Paraná, Curitiba, Paraná, Brazil; 4 Laboratório de Ecologia e Conservação, Departamento de Engenharia Ambiental, Setor de Tecnologia, Universidade Federal do Paraná, Curitiba, Paraná, Brazil; University of Waikato (National Institute of Water and Atmospheric Research), NEW ZEALAND

## Abstract

The increasing number of quantitative assessments of homogenization using citizen science data is particularly important in the Neotropics, given its high biodiversity and ecological peculiarity, and whose communities may react differently to landscape changes. We looked for evidence of taxonomic homogenization in terrestrial birds by investigating patterns of beta diversity along a gradient of human-altered landscapes (HAL), trying to identify species associated with this process. We analyzed bird data from 87 sites sampled in a citizen science program in the south Brazilian Atlantic Forest. Regional-scale taxonomic homogenization was assessed by comparing beta diversity among sites in different HALs (natural, rural or urban landscapes) accounting for variation derived from geographical distance and zoogeographical affinities by georeferencing sites and determining their position in a phytogeographical domain. Beta diversity was calculated by multivariate dispersion and by testing compositional changes due to turnover and nestedness among HALs and phytogeographical domains. Finally, we assessed which species were typical for each group using indicator species analysis. Bird homogenization was indicated by decreases in beta diversity following landscape changes. Beta diversity of rural sites was roughly half that of natural habitats, while urban sites held less than 10% of the natural areas’ beta diversity. Species composition analysis revealed that the turnover component was important in differentiating sites depending on HAL and phytogeography; the nestedness component was important among HALs, where directional species loss is maintained even considering effects of sampling effort. A similar result was obtained among phytogeographical domains, indicating nested-pattern dissimilarity among compositions of overlapping communities. As expected, a few native generalists and non-native urban specialists were characteristic of rural and urban sites. We generated strong evidence that taxonomic homogenization occurs in the south Brazilian Atlantic Forest as a result of a directional and nested species loss, with the resultant assemblages composed of few disturbance-tolerant birds.

## Introduction

Landscape changes due to human activities are the main cause of the recent biodiversity crisis [1], [2], [3]; the scale and magnitude of these changes has created a matrix of human-altered biomes, the so called anthromes of the Anthropocene [4]. The effects of landscape modifications are reflected in many aspects of biodiversity, altering natural community assembly processes in a typically directional, non-random manner [5], [6], [7], [8], although random processes can also occur [9]. One of the leading consequences of these negative impacts is biotic homogenization, i.e., the increasing similarity of biotas of large geographical areas over space and time, a multitaxa global phenomenon [7], [8], [10], [11], [12], [13]. A major process of human-induced alteration of landscapes is “urban sprawl”, which creates human-altered ecosystems very different from original ones, such as pastures, croplands and impermeable soil. Impacted landscapes lose original habitat, giving space to more homogeneous human-altered environments with respect to their spatial structure, abiotic conditions, biotic elements and, as a consequence, ecological processes [14]. Increasing attention has been given to urbanization as a principal source of biotic homogenization. For example, McKinney [15] found that plant community similarity from 18 state parks in the United States showed higher distance decay than urban plant communities in eight large cities of the country. Urban sprawl occurs concurrently with an increase in human population density, which is accompanied by significant negative impacts on biodiversity and ecosystem services [14], [15], [16]. Consequently, a greater understanding of how biodiversity patterns shift in sites immersed in different human-altered landscapes (HAL) is pivotal to support further actions on biodiversity management and conservation [17]. The effects of human-induced landscape changes on biodiversity have been examined in a number of taxa, among which birds are one of the most widely known. Taxonomic homogenization has been particularly well assessed (*e*.*g*. [18], [19], [20]), but there is an enormous bias towards Neartic and Paleartic assemblages. In contrast, the few studies of urban birds in Latin America have usually addressed descriptive goals, such as the generation of species lists for urban avifauna [21]. Larger scale and process-based studies are lacking, even though the Neotropical realm houses the most diverse bird community worldwide. Meanwhile, species extinction in mega-diverse tropical areas is disproportionally high compared to temperate regions [22], [23], and a possibility is that tropical species are very sensitive to human disturbance [24] and may present ecological patterns not shared by temperate counterparts [25]. Brazil, the largest country in the Neotropical realm, is rapidly accumulating empirical evidence concerning the impacts of human-induced landscape modifications on biodiversity [8], particularly in the Brazilian Atlantic Rainforest [26], [27], [28]. Still, Brazil lacks published data accessible for macroecological research and usually only few sites are included in global analyses; this was illustrated by two recent studies that included only a single Brazilian site in their dataset [11], [29]. As ecosystem services are continuously degraded, especially in and around tropical urban areas [30], the identification of patterns in diversity shifts can support further investigations into the mechanisms of causation, allowing the development of better management practices [31]. At the same time, considering the costs of large scale sampling in megadiverse countries both in space and time, citizen science initiatives are especially powerful tools to help survey and monitor biodiversity [32], [33].

In the present paper we evaluate taxonomic homogenization of bird assemblages along a gradient of human-altered landscapes at a regional scale in the south Brazilian Atlantic Rainforest, one the most threatened global diversity hotspots. We use data gathered through a newly formed citizen science initiative to extract beta diversity values from sites surveyed by enthusiasts, while accounting for influences in dissimilarity due to geographical distance and zoogeographical affinities. We primarily follow the analytical framework proposed by Anderson and co-authors [34]. However, since analyses based on broad-sense similarity measures can lead to misinterpretations of the homogenization pattern [11], we further decompose beta diversity into its turnover and nestedness components, each reflecting different ecological aspects [35], [36]. Dissimilarity among sites–as a direct measure of beta diversity [37]–is influenced by species replacement (the turnover component) and by “dissimilarity associated to species losses in which an assemblage is a strict subset of another” (the nestedness component; [38]). As the sum of both components result in global beta diversity, the relative importance of each metric is useful to understand which ecological processes are responsible for the observed patterns [39]. Regarding taxonomic homogenization, urban sprawl can be represented by a subset of species present in richer ones (i.e. nestedness caused by the fact that disturbance-tolerant species should persist in altered landscapes, while sensitive forest specialists are extirpated), albeit with important replacement contributions (i.e. turnover caused by the fact that open-habitat species should colonize these landscapes, while being scarcer in landscapes with more forest cover). Such a nested-patterned loss of species from unaltered into modified landscapes should be reflected in a higher importance of nestedness-related dissimilarity in differentiating bird species composition [8], [39], but only among HAL categories. By disentangling the importance of both metrics we thus aim to elucidate patterns and causes of the homogenization process.

## Methods

We used the bird dataset of a citizen science initiative [40], the Participative Inventory of the Birds of Paraná (IPAVE) to select 124 sites surveyed during one week in September 2012 in the state of Paraná, southern Brazil (S1 Supporting Information). In this initiative, birdwatchers, photographers, students and professional ornithologists were instructed into protocol standardization for data sampling, and to identify species correctly. Also, all sampling were checked to avoid inconsistencies considering each species’ distribution range and habits. Therefore, we believe that data is reliable to evaluate biodiversity patterns. Each site was classified into one of three HAL categories of increasing impact following an urban sprawl gradient, from sites with predominantly natural vegetation cover, through rural areas and urban sites. This assessment was performed by visual analysis of satellite images of each site’s surrounding landscape (S1 Supporting Information). Additionally, each site was classified according its phytogeographical position, identified from a regional map of the state’s vegetation [41]. These were divided into three major categories: dense forest, mixed forest, and semideciduous forest, each of which is known to house specific avifaunal assemblages [42].

Records from sites that were very close together (< 0.5 km) were pooled, producing a new set of 114 samples spanning all of the state’s territory (S1 Supporting Information). We then computed the first quartile of terrestrial bird species richness for these samples, and sites whose richness was lower than this value (Q_1/4_ = 30) were excluded from our sample pool (n = 27). This reduced the data set to 87 sites for subsequent analysis (number of HAL sites: 26 natural, 35 rural and 26 urban; number of phytogeographical category sites: 17 dense, 28 semideciduous and 42 mixed; details in S1 Supporting Information). We used this conservative criterion to increase reliability of our data, ensuring that differences among sites were not due to a large number of false absences. Indeed, we are confident that the sites studied here, even urban ones, should have more than 30 species [43]. While we acknowledge that excluding sites with less than 30 species may represent an exclusion of species-poor sites instead of an exclusion of sites that were under-represented, we decided to be conservative to avoid under-representation errors. Also, we highlight that species-richness range in our dataset is still high, ranging from 30 to 173 in the poorest and richest sites, respectively. Another consequence of using data from multiple observers could be attributed to sampling effort, and in order to address this issue we performed a generalized linear model (GLM) using Poisson distribution [44] with effort and the two factors of classification (HAL and phytogeographical categories) as explanatory variables to assess whether species richness differences was maintained independently of sampling effort. We found that richness was different among HALs and phytogeographical groups, independent on sampling effort (S1 Supporting Information). Indeed, although sampling effort influences species richness, this factor had the lowest coefficient estimation, showing that any of the categories contribute more to species richness differences than sampling effort alone. Finally, it is notable that species richness is significantly higher in natural sites in the dense forest, and that urban areas had the lowest richness (S1 Supporting Information). Subsequently, we tested for spatial autocorrelation in our data, a second potential source of bias that must be taken into consideration since sampling sites were not randomly selected across HALs and phytogeographical categories. Our results showed that, at the spatial scale considered, compositional dissimilarity is independent of geographic distance in both categories evaluated, thus allowing us to rule out significant effects of spatial autocorrelation in our analysis (S1 Supporting Information).

It could be argued that the temporal restriction of IPAVE can underestimate local bird richness (alpha diversity in each site), especially since some observations span only a few hours of sampling effort (S1 Supporting Information). However, since most communities are composed of a few frequently recorded species, exhaustive inventories tend to increase the number of rare and vagrant taxa, which can substantially cloud the assessment of changes in the dominant species, and is especially influential on analyses based on incidence data, such as ours [45]. Furthermore, in order to obtain meaningful data of biodiversity responses to environmental changes, performing spatially extensive sampling has been demonstrated to be more effective than intensive efforts on fewer sites [46]. We therefore believe that the IPAVE protocol resulted in an overview of the state’s bird distribution, providing a snapshot of bird assemblages found in each visited site. We are confident that the protocol was able to estimate both the most common species and also some rare. Since our purpose was to identify general patterns in community homogenization, the dataset provided by IPAVE is ideal for our proposed analysis.

We used multivariate dispersion as a measure of beta diversity [34], [47] to reflect the level of avifaunal homogenization. This metric is the average distance of the sampling points to the group centroid in an ordination. Groups were defined according to the specific categories of HAL (natural, rural, urban) and phytogeographical class (dense, semideciduous, mixed). Sites with more similar avifaunal composition are represented by points closer together in the ordination space, and groups with the lowest mean value of distance to the centroid represent more homogeneous assemblages.

The ordination technique used is the Principal Coordinate Analysis (PCoA), which is based on a dissimilarity matrix. Consequently, the choice of the distance index is crucial. Classical incidence indexes, such as the Jaccard dissimilarity index, can result in biased similarity values, especially when alpha diversity between sites is very different [48]. Indeed, our data include sites with richness ranging from 30 to 173 species, which could result in the underestimation of dissimilarities. To avoid this effect on our analysis we chose the Raup–Crick index of dissimilarity [37], which performs random permutations to determine how often a comparable level of similarity occurs, considering the available species pool and each site’s richness [49]. For these analyses we used the *betadisper* routine within the *vegan* package [50] for R software [51]. The original implementation of this metric [49] does not constrain for species incidence, but rather assumes species would be sampled with equal probability from the entire species pool. To overcome this issue we constructed the dissimilarity matrix using the *raupcrick* function, which defines the probability of selecting species as being proportional to the species’ frequencies in each category [48]. Differences in pairwise multivariate dispersions of the groups were assessed with the function *permutest* with 9999 permutations. We plotted the resulting ordination of the full data matrix to visualise compositional differences and point dispersal in space (beta diversity) by grouping sites to both HAL and phytogeographical categories. This ordination was performed in Past 3.01 [52].

We further decomposed beta diversity into turnover and nestedness components to evaluate the relative importance of each in creating dissimilarity patterns among categories [35], [38]. For this analysis, we used the pairwise Jaccard dissimilarity index [36] with the function *beta*.*pair* within *betapart* package [53] for R software [51], which provides three dissimilarity matrices, one for each of the beta diversity components computed (β_jac_, β_jtu_, β_jne_). Given that the goal was to compare these components among HALs and phytogeographical categories, the bias considering a general Jaccard index explained above did not affect the output of the analysis (i.e., β_jne_ explicitly demonstrate differences in composition given species richness, while β_jtu_ is independent of richness [38]). Also, there is no decomposition of beta diversity of the Raup-Crick index implemented in R. In this analysis, if species composition differences are explained by the nestedness component, it follows that a non–compensatory loss of species is responsible for the differentiation of assemblages in each category. We acknowledge that other frameworks could be used to quantify species loss among HALs and phytogeographical categories [54], [55], and that these methods are the subject of continuous discussions in the literature [38], [54], [55], [56], [57], [58]. However, considering our specific focus on how to evaluate beta diversity patterns, we decided to proceed within Baselga’s approach. We tested whether we could detect significant compositional changes due to turnover and nestedness in both HAL and phytogeographical categories, while simultaneously accounting for differences in sampling effort, with PERMANOVAs for each of the components [59] with 999 permutations, using function *adonis* in *vegan* [50].

Finally, we identified which bird species characterized each HAL class and each phytogeographical category by means of indicator species analysis [60]. This technique considers each species’ habitat fidelity (number of individuals; in this case, the frequency of occurrence among sites within each category) and specificity (patterns of presence–absence among categories) to calculate a percentage indicator value (IndVal). For a species to be considered characteristic of a certain category, it must have been found reliably (i.e. high frequency of occurrence) and almost exclusively within that category. We only considered species that had IndVal statistically significant (p < 0.05) following Dufrêne & Legendre’s [60] random reallocation procedure. We acknowledge that IndVal does not consider differences in sampling effort, and we could use a GLM approach to evaluate relationships between species and HALs and phytogeographical categories. However, we decided to keep this analysis for three reasons. First, GLM does not have an indicator purpose, while IndVal was proposed to formally test this aspect. Second, sampling effort was found to be the least important predictor of species richness differences in our data (see Supplementary Information). Finally, species pointed out as important indicators according to IndVal was in accordance with our expert knowledge, considering the disturbance-tolerance continuum and zoogeographical affinities (see Results and Discussion). This analysis was performed using function *indval* in the *labdsv* package [61] for R software [51].

## Ethics Statements

The authors declare that no special requirements were needed to access public sites sampled by volunteers during the 2012 study. All private land owners were contacted by volunteers prior to bird sampling, as encouraged by the IPAVE coordinators, but no specific permits were provided for visiting sites. Some protected areas were included in sampling, but none of which required special permits by environmental authorities. Protected and/or endangered species sampled during IPAVE were not subjected to any form of capture stress, since records consisted of visual or aural cues recorded by the volunteers. Any questions regarding ethical issues of the IPAVE project [40] can be directed to the corresponding author, who is also part of the coordinator team.

## Results

**Fig 1:**
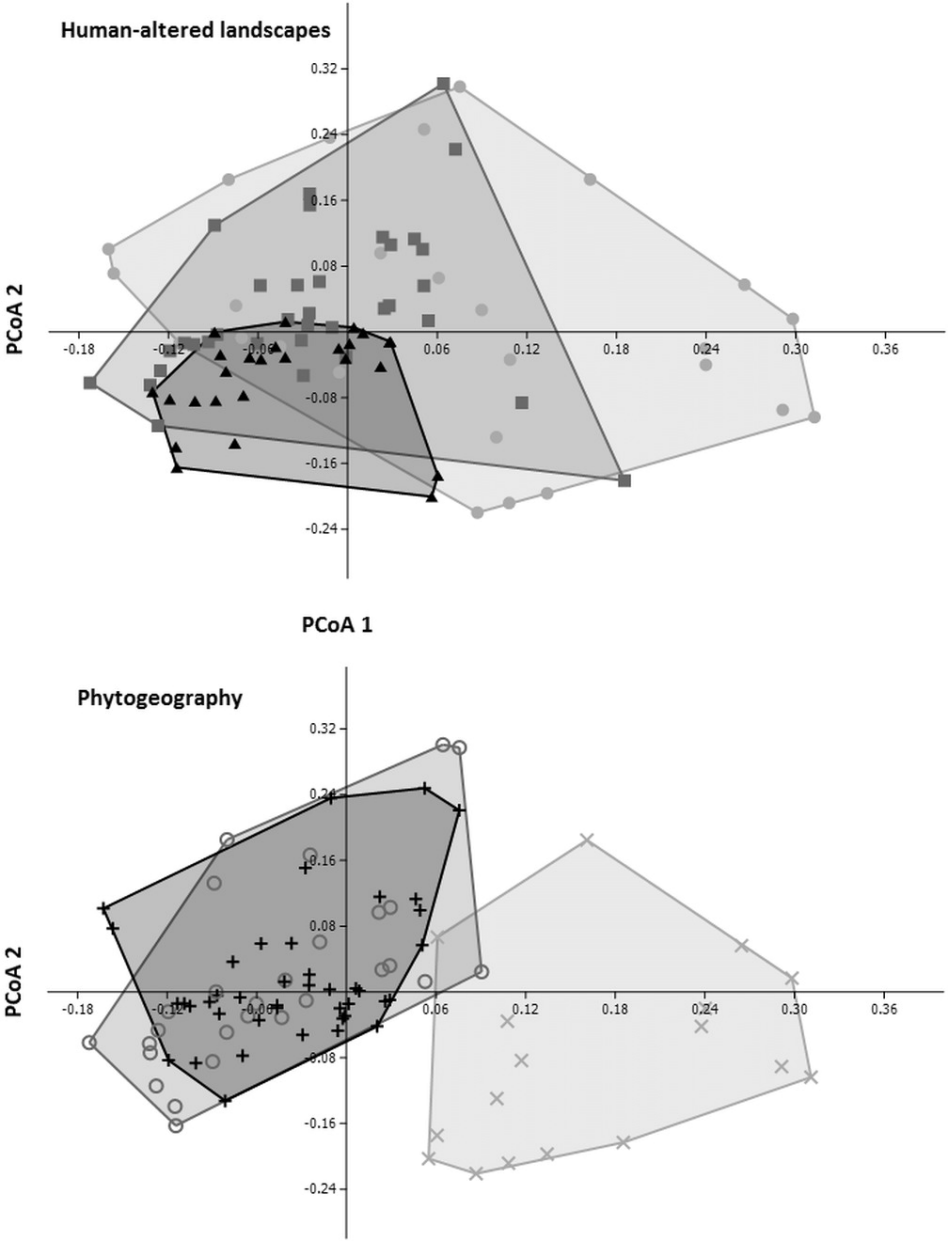
Scatter plot depicting the first two axes of the ordinations of bird species composition. Principal coordinates analysis (PCoA) was computed using the Raup–Crick method. The axes represent 11,1% and 8,6% of data variation, respectively for PCoA 1 and 2. Each point represents the composition of the avifauna at a particular sampling site; point dispersal in this two dimensional space corresponds to total beta diversity within each category (demarcated by convex hulls). The influence of human-induced alterations of the landscape on the composition of the assemblage (above) appears to show a nested pattern, with rural and urban birds representing a subsample of the avifauna of the natural sites. Total beta diversity is clearly lower in urban sites (black triangles) compared to both rural (dark grey squares) and natural sampling sites (light grey dots). Bird species composition is also influenced by phytogeography (below), with the dense forest (light grey “x”) avifauna considerably different from that of both the mixed forest (black “+”) and the semideciduous forest (dark grey “o”); beta diversity (point dispersion in space) in the mixed forest is lowest among the three categories.

The PCoA ordination revealed divergence in the avifaunal composition among the HAL categories, consisting of a gradual shift from natural to rural to urban sites (Fig 1). This pattern points to the presence of some exclusive or specialist species in rural and urban landscapes that are either not present or less frequent in more pristine sites. We also found segregation between dense forest birds and a cluster of both semideciduous and mixed forest birds, indicating dissimilar species compositions for these groups, influenced by species turnover (Fig 1).

**Fig 2:**
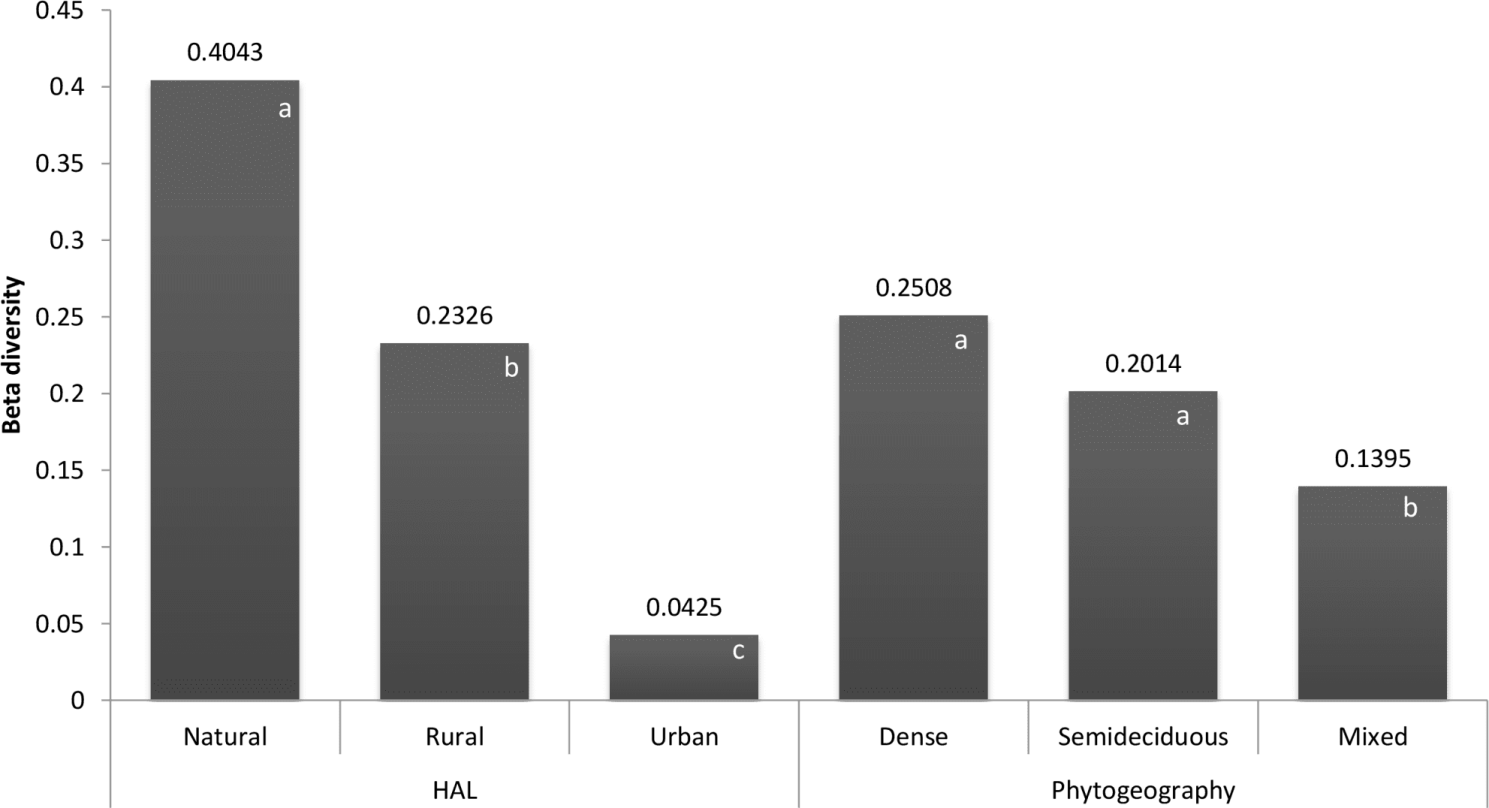
Bird beta diversity in each group of sites from human-altered landscape (HAL) and phytogeographical categories. Average distance to centroid in each group of sites from human-altered landscape (HAL) and phytogeographical categories, as obtained from a multivariate homogeneity of dispersion analysis. This metric corresponds to the beta diversity value used to assess the degree of homogenization of Atlantic Forest terrestrial bird assemblages. Statistically significant differences in the mean values of the distance to the centroid are depicted by different letters inside each column.

We found consistent differences in beta diversity between HAL categories (Fig 2; distance to centroid between groups ANOVA F = 47.45, P < 0.0001): natural sites have higher beta diversity than rural sites (P = 0.001), and urban bird faunas proved to be the most homogeneous category (P = 0.001). That is, the average dissimilarity of sites from their group centroid in multivariate space is significantly higher in natural than in rural and, successively, urban sites, suggesting a directional homogenizing shift in bird assemblages along the human-induced alteration of landscape gradient. Beta diversity of rural sites was roughly half of the beta diversity of natural habitats, while urban sites held less than 10% of the natural areas’ beta diversity. Bird beta diversity between different phytogeographical domains was also different (Fig 2; distance to centroid between groups ANOVA F = 5.0828, P = 0.009), but this result was attributable to the larger compositional similarity among mixed forest birds when compared to both dense (P = 0.001) and semideciduous (P = 0.034) sites, which had indistinguishable beta diversity values (P = 0.31).

Analysis of bird composition shifts based on the Jaccard dissimilarity indexes (β_jac_) showed differences in species composition with respect to the interaction of HAL and phytogeographical categories (PERMANOVA F_interaction_ = -12.341, P = 0.018), a result similarly found in the species turnover component (β_jtu_; F_interaction_ = 13.669, P = 0.036). Nestedness-related dissimilarity (β_jne_) was accountable for differences in the composition of bird assemblages in two situations: considering the interactions of sampling effort and HAL categories (F_interaction_ = 40.962, P = 0.04), and the interaction of sampling effort and phytogeographical categories (F_interaction_ = 44.849, P = 0.027). These results indicate that nested-patterned compositional differences among bird species found in natural, rural and urban sites, and also among assemblages of dense, semideciduous and mixed forest species, depends on sampling effort. Such differences arise as birds assemblages in each category represent a subset of those found in another category; in turn, these patterns can be attributable to directional species loss in the rural and urban sites relative to natural sites, a pattern that is maintained even when considering the effects of sampling effort (see also Fig 1). Interpretation of these patterns amongst phytogeographical domains is not straightforward, but do indicate nestedness among compositions of overlapping communities of mixed and semideciduous forests as shown in Fig 1.

IndVal analysis revealed that 109 species were typical of natural sites, a single species was associated with rural sites and 12 species were characteristic of urban settings. Among the phytogeographical groups we identified 69 species characteristic of dense forest sites, 12 species characteristic of semideciduous forests and 15 species characteristic of mixed forests (S1 Supporting Information). IndVal was highest for the dense forest phytogeographical realm, with values reaching up 0.824 (*Tangara cyanocephala*). In the HAL groups, IndVal was more modest, the highest value being 0.519 in the natural group (*Crypturellus obsoletus*) and 0.456 in urban sites (*Passer domesticus*).

## Discussion

We provide a quantitative evaluation and strong evidence for biotic homogenization driven by human-induced alteration of landscapes. Most importantly, these findings enhance our understanding of the effects of human impacts on Neotropical birds, in particular, in one of the most threatened diversity hotspots in the world [26]. Additionally, we argue that the use of citizen science results in a megadiverse country is one of our study’s greatest strengths. These initiatives are more established in Europe and North America [62], [63] and their potential to uncover macroecological patterns is unquestionable [64]. In the Neotropical realm and particularly in Brazil, where sampling by individual scientists or organizations at large scales is almost impossible [32], such initiatives have only recently begun, but in just a few years they have already demonstrated their great potential (*e*.*g*. [65]). Our results suggests that even when sampling effort variation among citizen scientists data are large, general ecological patterns are still unveiled; in our case, bird diversity patterns among human-altered landscapes–where urban sites are species-poor areas, mainly composed of birds already present at rural or natural settings–and among phytogeographical domains–where sites of dense forest harbor richer bird communities, composed of markedly different species than those found in mixed or semideciduous forests. Citizen science initiatives such as IPAVE must be encouraged and continuously enhanced, with the output data rigorously vetted so it can be used in multiple ecological research areas. These datasets are increasingly important to improving our general understanding of biodiversity distribution patterns, especially in developing countries where anthropogenic pressures are enormous and the implementation of sound management plans is urgent. Despite sampling limitations that precluded the development of a full representation of the avifauna in natural areas, we were able to generate robust and clear patterns of biotic homogenization. Increasing the number of rare species, by increasing sampling effort, will only strengthen the effective size of human-induced biotic homogenization, since more rare species will be probably found in natural areas [66].

Although we did not analyze changes in species composition along a temporally explicit gradient, our results do reveal homogenization patterns across space, usually considered to be “only tentative evidence” of this process [67]. However, present-day landscape patterns can be used as temporal surrogates if we assume that land use intensity by human settlement and urban sprawl follows a gradient from little altered habitats, to rural and urban areas [18]. This process embraces the temporal dimension implicitly (i.e. space-for-time substitutions), as has been used in several studies that assessed the urbanization gradient [18], [20]. Indeed, landscape occupation *per se* is often closely related to homogenization [12].

Effects of human-induced landscape changes on biodiversity have been examined in a number of taxa, among which birds are one of the most widely known [8], [18], [21], [68]. These studies found consistent trends in bird alpha diversity (species richness), abundance patterns and community composition worldwide. Species richness increases in certain intermediately disturbed situations while it decreases in heavily urbanized areas [15], [18], [69], [70], [71]; the loss of species in rural and urban sites relative to natural areas was also observed in the present study. Abundance patterns are also affected, with some urban areas harboring more abundant bird assemblages, mainly due to the increased dominance of a few species [7], [15], [21], [72], [73]. Species composition, on the other hand, seems to be affected in a more predictable manner, with the exclusion of rare and specialized species accompanied by the colonization and spread of few generalist and non-native birds [7], [8], [20], [45], [69]. The potential for each species to adapt to anthropogenic disturbances results in species-filtering of remnant assemblages, which is in turn dependent on certain phenotypic traits, and these patterns are the basis of the ‘urbanization tolerance hypothesis’ [74]. This non-random species-loss leads to biotic homogenization across several scales and taxa [8], [12], [28], [31], [75], and often reduces biodiversity by decreasing native species richness and, eventually, increasing non-native importance [10], [12], [48].

In accordance with such predictions, continental analyses of birds found reduced dissimilarity following increased anthropogenic disturbance in North America [45]. Similar results were obtained when the avifauna in different cities was compared to bird assemblages in adjacent natural habitats across Europe [71], [76]. Here, when compared to natural sites, the decline in the dissimilarity of terrestrial bird assemblages was largest among urban areas, which lost nearly 90% of their original beta diversity; rural sites also had a substantial decrease in their beta diversity (to roughly 50% of the beta diversity of natural areas, see also Fig 1). In line with other studies outside of North America and Europe [19], [20], [24], [77], [78], [79], [80], our results add empirical support to the hypothesis that bird assemblages outside temperate zones are subjected to a large decrease in beta diversity due to urban sprawl, despite the rich avifauna that can be found in large cities on city-wide scales [81], [82], [83].

The compositional shifts found in our study agree with predictions based on expected changes in species assemblages following changes in land use, from pristine to agricultural lands and urban habitats [8], [18], [19], [74], as well as those based on zoogeographical affinities of the south Brazilian avifauna [42]. Spatial variation in beta diversity among the HALs reflects the homogenization of terrestrial bird assemblages, as measured by the dissimilarity of assemblages, a procedure widely applied in the homogenization literature [7], [67]. By explicitly assessing the relative importance of the turnover and nestedness components of beta diversity, our study fills an important gap in understanding patterns of homogenization on the Neotropical realm.

Our results also illustrate the importance of nestedness in homogenization assessments. Alterations in species composition among natural, rural and urban sites were a result of both true species turnover and derived from species-loss patterns. Nestedness-related dissimilarity accounted for compositional differences among HAL categories, even accounting for variation in sampling effort effects, reinforcing the paradigm of directional, non-random, loss of species following human disturbances [8], [20], [28], [31], [71], [84]. Simultaneously, dissimilarity due to true species turnover indicates that some species act as “winners” in human-altered habitats, exploiting novel ecosystems in regions that were otherwise inhospitable [7], [85]. Differentiation of dense, semideciduous and mixed forest avifaunas due to turnover reflects the natural geographical replacement of species, as expected from known patterns. Taken together, beta diversity patterns unveiled by these analyses clearly reflect the effects of taxonomic homogenization due to anthropogenic impacts.

The nestedness component was also responsible for assemblage differences among phytogeographical domains, when in conjunction with sampling effort. While this may seem to contrast with our initial expectations, the influence of sampling effort on both categories analyzed may have a clear biological correlate. Sampling effort is expected to be influential on nestedness-related dissimilarity among assemblages because it affects species richness (see GLM results). Thus, sites with more effort dedicated to sampling should include most of the species found in lesser-sampled sites (i.e. common species) in addition to species not usually found there (i.e. rare species). This phenomenon is a known property of sampling ecological communities, and is explicitly assessed in Baselga’s nestedness component [38].

On the other hand, the turnover component is not expected to be much affected by sampling effort, since effort should not be biologically relevant to sample species substitution in space (as spatial distribution preferences is not related to species commonness). Indeed, our results point that this is the case. Finally, mixed results in the general Jaccard index, when summing both components, is also a probable result, but still the interaction among our categorical factors was shown to be a better predictor of assemblage composition differences, regardless of sampling effort differences, a result similar to that found in our GLM.

Indicator species analysis revealed that species associations among HAL categories are consistent with our expectations. The sheer number of species characteristic of each category suggest that natural sites (n = 109) are home to a plethora of specialists, while rural (n = 1) and urban sites (n = 12) are preferentially occupied by few “winner” birds, probably generalist birds that were only able to thrive due to changes in the biotic and abiotic environment, where more specialized taxa were excluded [7], [19]. Of the 109 species associated with natural sites, 83 are forest dwellers, (see also S1 Supporting Information), i.e., birds inhabiting the forest interior that are usually extirpated from fragments subjected to human disturbance, an indicator of specialization.

The homogenizing effect of landscape changes on Atlantic forest birds is thus derived from a pattern where assemblages in rural and urban sites represent a subset of the surrounding natural species pool [71], [86]. As evidenced by indicator species analysis and in agreement with studies elsewhere [20], [29], [31], [76], native generalist species provided the largest contributions to the blending effect found here, while non-native birds were not of paramount significance in the homogenization of Atlantic Rainforest avifauna. A single species, *Trogon surrucura*, was associated to rural sites, albeit with a low IndVal (0.287), possibly because it is also commonly found in natural and urban forest remnants [83]. This is a forest frugivore with medium sensitivity to disturbance [87] that is found in small and isolated forest patches and is usually one of the few remnant frugivores found in disturbed areas [88]. Urban-associated species include two non-native urban specialists, the house sparrow (*Passer domesticus*) and the domestic pigeon (*Columba livia*), two of the most widespread synurbic birds worldwide [86], and two additional non-native birds that frequently colonize semi-natural areas around cities, the common waxbill (*Estrilda astrild*) and the blue-fronted parrot (*Amazona aestiva*). In addition, several open area species were also associated with urban sites, including two generalist insectivores, the yellow-browed tyrant (*Satrapa icterophryus*) and the large elaenia (*Elaenia spectabilis*), a single generalist aerial species, the blue-and-white swallow (*Pygochelidon cyanoleuca*), and birds that are currently expanding their range following deforestation, the rufous hornero (*Furnarius rufus*), the swallow-tailed hummingbird (*Eupetomena macroura*), the eared dove (*Zenaida auriculata*), the shiny cowbird (*Molothrus bonariensis*), and the campo flicker (*Colaptes campestris*). This result is similar to those found in many urbanization studies, where insectivorous generalists benefit in more urbanized areas [19], [89] (but see [90], [91]), including in the Neotropics [70], [79], [80].

Among the many outcomes of this “winner-loser replacement” are the ecological disturbances that some benefited native species can cause. Acting in synergy with the more obvious impacts of habitat loss and fragmentation, such species can promote a rearrangement of assemblages. A single species’ strong aggressive behaviors may cause major ecological disruptions, as exemplified by the noisy miner (*Manorina melanocephala* Latham, 1802) in Australia [92]. In the present study two species could be classified as agents of disturbance: the swallow-tailed hummingbird and the shiny cowbird. Both species are well-known “urban adapters” that are expanding their geographical range following landscape changes. The first species is the largest hummingbird in the Atlantic Rainforest and may be outcompeting many other nectar feeding birds because of its high resource intake [93], feeding plasticity [94], and aggressiveness [95], being usually dominant in interspecific interactions [96]. The shiny cowbird impacts other birds by increasing nest parasitism in human-altered habitats, as has been observed in Brazilian savannas [97], [98], [99]. Nest parasitism can lead to regional scale impacts, as documented by the congeneric brown-headed cowbird *Molothrus ater* (Boddaert, 1783) [100], [101]. The supposed low effect of non-natives on more altered sites is, however, a taxonomic interpretation of impact. Non-natives may play a disproportional role in the local ecology and synergistically impoverish native species assemblages in human-altered landscapes [75], [102]; local studies should be conducted to evaluate these interactions.

The replacement of species among phytogeographical categories found in the present study agrees with our expectations. Not surprisingly, many of the bird species associated with each domain were expected based on known zoogeographical patterns (*e*.*g*. *Tangara cyanocephala* for dense forest; *Aratinga leucophthalma* for semideciduous forest; *Leptasthenura setaria* for mixed forest [42]). PERMANOVA and PCoA results also reveal that the dense forest avifauna is quite different from the avifauna found in semidecioduous and mixed forests, a finding reinforced by the high number of birds associated with dense forests (n = 119) and lower numbers of species characteristic of semideciduous (n = 30) and mixed forests (n = 22). Interestingly, beta diversity of mixed forest assemblages is lower than that found in both dense and semideciduous areas (Fig 1). This pattern seems to be independent of the spatial extent of mixed forests, given that mixed forests form the second largest phytogeographical domain in the state. Previous studies have found that even small mixed forest remnants harbor rich bird assemblages, composed of many species that seem to use edge and forest interior indifferently [103, [104]. For instance, *Araucaria* plantations–the dominant tree characterizing mixed forests–house sensitive and endemic birds associated with this forest type [105], [106]. A reasonable explanation for this pattern is the preadaptation of mixed forest avifauna to such disturbance, so that species loss due to this source of anthropogenic impact is lessened by the birds’ ability to live in secondary and/or disturbed forest fragments, hence diminishing dissimilarity in natural, rural and even urban areas. Indeed, *Araucaria* forest paleodynamics suggest that forest vegetation was restricted to river valleys immersed in a grassland-dominated landscape, and began a rapid expansion into open areas during the late Holocene, mainly along riparian strips [107] (and references therein). Collectively, these results pose an interesting research agenda to investigate the mixed forest birds’ ecological traits, since a more general phenomenon may be leading to the observed pattern.

In summary, our results highlight the occurrence of biotic homogenization due to human-induced alterations [31]. The non-random filtering of species that thrive in HALs decreased beta diversity on a regional scale [108], [109], thereby promoting the homogenization of terrestrial bird assemblages in southern Brazil, a result similar to those found in other parts of the Atlantic Forest [28] and in the Brazilian Amazon [8]. Anthropogenic disturbances reinforce deterministic processes in community assembly and the resulting assemblages are a non-random subset of the regional species pool [23], [28], [31], [86]. Our results also reinforce the idea that generalist, broadly distributed, open-area native species are more prone to colonize and thrive in human-altered environments, as well as cosmopolitan non-native species, supporting the ‘urbanization tolerance hypothesis’ [18], [74], [76], [110], [111]. Rural areas probably act as a middle step on a gradient of species replacement [8], [109], [112] with few species especially associated with these landscapes. Human-altered habitats, often referred to as novel ecosystems, are increasingly dominating the world’s landscapes, and their potential to support biodiversity is seriously questioned [113]. Based on these collective results we can foresee that HALs will increasingly promote the dominance of disturbance-adapted native species worldwide, at the expense of rich specialist assemblages, thus promoting negative impacts on biodiversity and ecosystem services [14], [31]. In a context where humanity has transgressed Earth´s operational limits in terms of biodiversity change [114], where HALs and especially urban expansion will increase, urban planning urgently needs to become more ecologically sensitive, and take into account all biotic components.

## Supporting Information

S1 Fig (TIF)

S2 Fig (TIF)

S1 Supporting Information (DOC)

S2 Supporting Information (XLSX)
