# Supplementary figures and images for: Human-Induced Landscape Changes Homogenize Atlantic Forest Bird Assemblages through Nested Species Loss
Source: PLoS One. 2016 Feb 3;11(2):e0147058. doi: 10.1371/journal.pone.0147058 (PMC4739515; doi:10.1371/journal.pone.0147058)

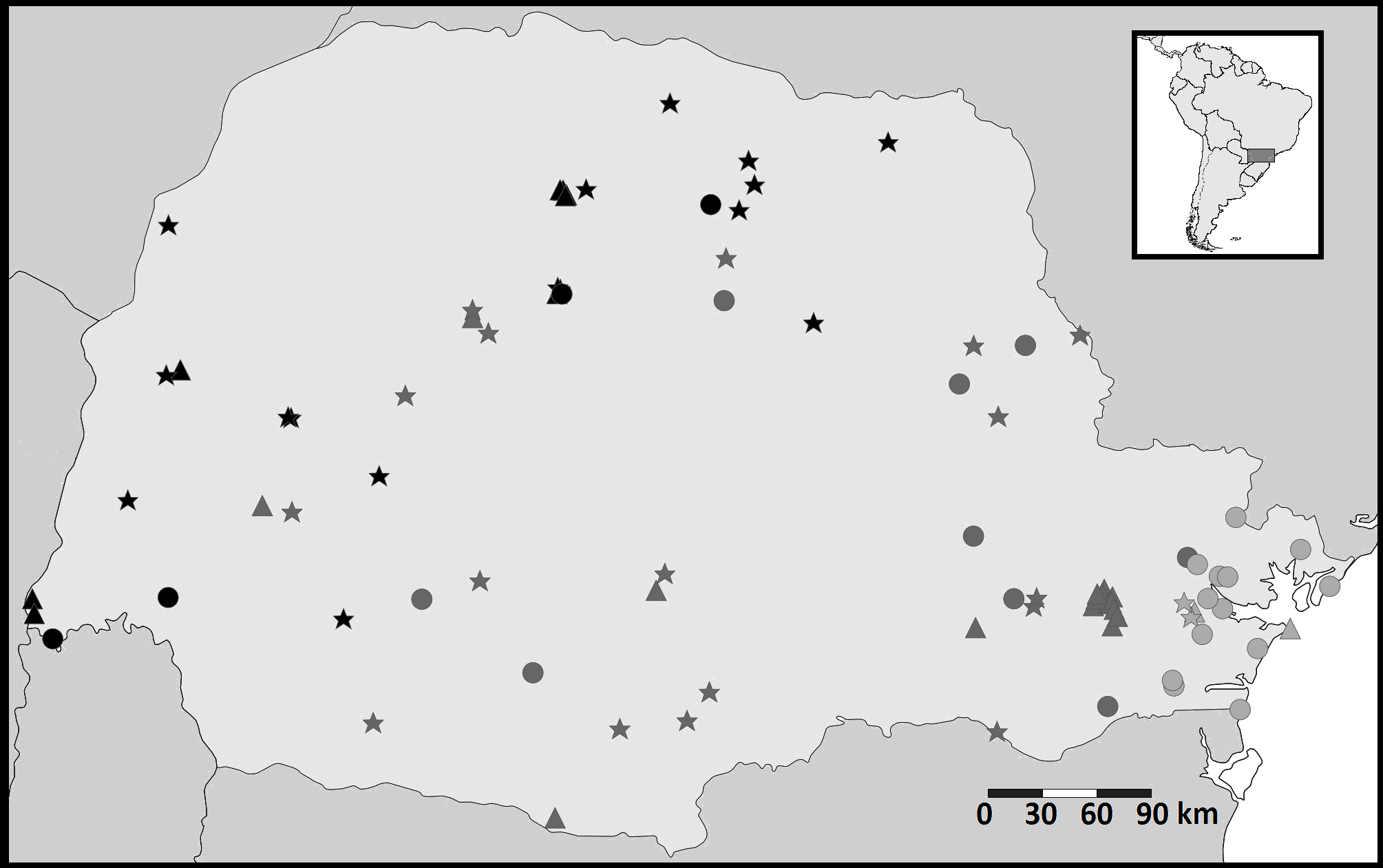

Supplement: S1 Fig — (TIF) [file pone.0147058.s001.tif]

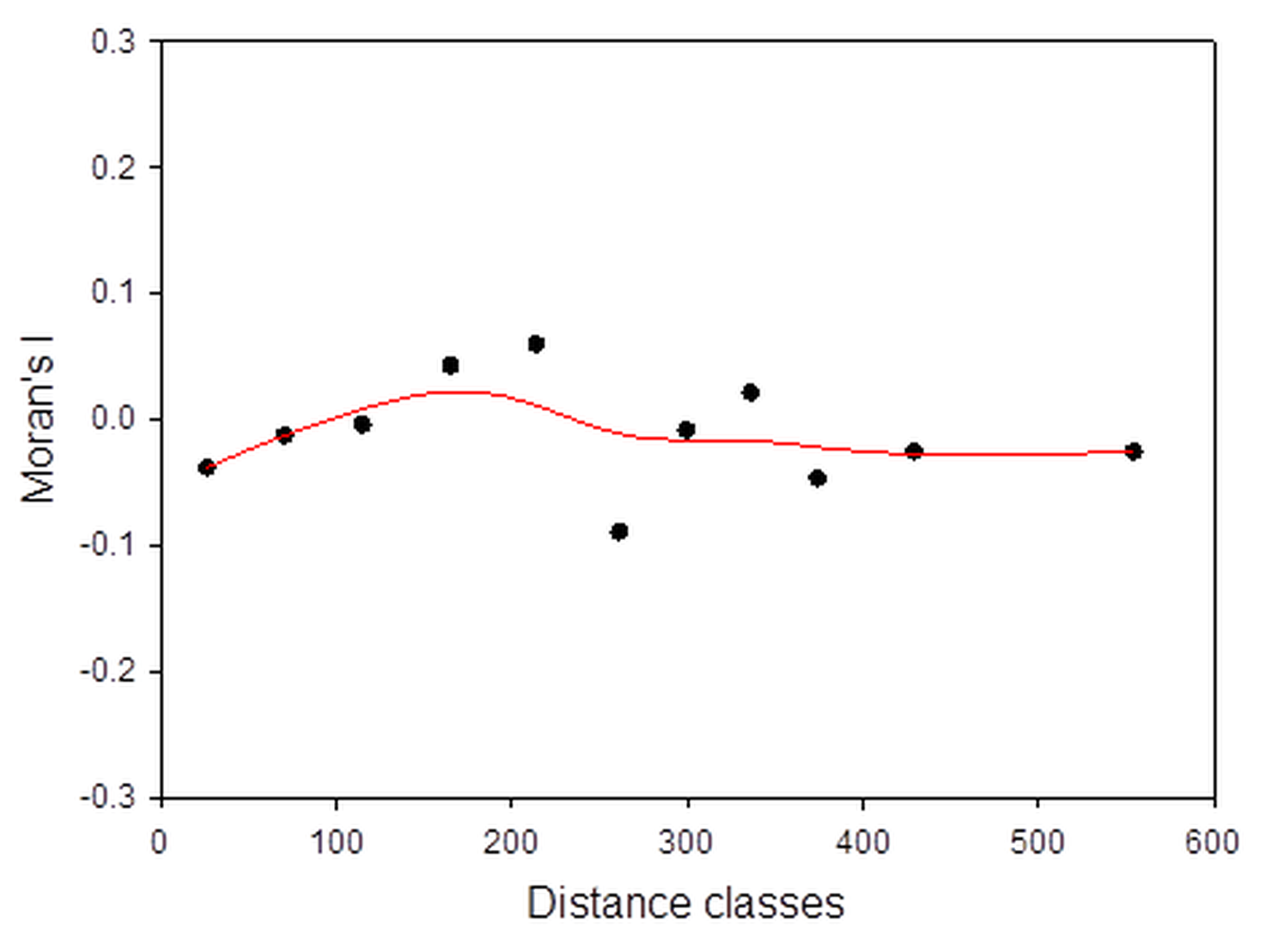

Supplement: S2 Fig — (TIF) [file pone.0147058.s002.tif]
