# Supplementary material for: Human-Induced Landscape Changes Homogenize Atlantic Forest Bird Assemblages through Nested Species Loss
Source: PLoS One. 2016 Feb 3;11(2):e0147058. doi: 10.1371/journal.pone.0147058 (PMC4739515; doi:10.1371/journal.pone.0147058)
Supplement: S1 Supporting Information — (DOC) [file pone.0147058.s003.doc]

Human-induced landscape changes homogenize Atlantic Forest bird assemblages through nested species loss

Marcelo Alejandro Villegas Vallejos^1^*, André Andrian Padial^2,3^, Jean Ricardo Simões Vitule^3,4^

^1^ Hori Consultoria Ambiental, Curitiba, Paraná, Brazil.

^2^ Departamento de Botânica, Universidade Federal do Paraná, Curitiba, Paraná, Brazil.

^3^ Programa de Pós-Graduação em Ecologia e Conservação, Setor de Ciências Biológicas, Universidade Federal do Paraná, Curitiba, Paraná, Brazil.

^4^ Laboratório de Ecologia e Conservação, Departamento de Engenharia Ambiental, Setor de Tecnologia, Universidade Federal do Paraná, Curitiba, Paraná, Brazil.

* Corresponding author

E-mail: mvillegas.bio@gmail.com

# S1 Supporting Information. Extended Methods.

*Study area.* The dataset used in the present analyses was acquired in the state of Paraná, southern Brazil, which is located between latitudes 22°29’30”S and 26°42’59”S; and longitudes 48°02’24”W and 54°37’38”W (S1 Fig.). Since most of the state’s territory lies south of the tropic of Capricorn, Paraná is mostly governed by subtropical climates, locally influenced by geographical features. The state is roughly divided into the coastline and the inner plateaus, which are limited in eastern portion of the state (around 48°58’W) by the massive Serra do Mar mountain range [1]. The relatively narrow littoral has a climate much influenced by the sea, with higher mean annual temperatures and rainfall, which is reflected in its luxurious tropical and subtropical dense forests distributed across a large altitudinal range, from sea level up to 2000 m. To the west of the Serra do Mar, the higher elevations of the plateaus – non-rarely reaching altitudes above 1000 m – define a more clearly subtropical climate, with cooler winters and moderate mean annual temperatures. In a very simplified form, the plateaus are higher in the east and south with gradually decreasing altitudes towards the west and north-west, these with comparatively higher temperatures. This configuration is also reflected in the plateaus’ vegetation: higher altitude areas, mainly at the center and south of the state, house subtropical mixed forests and natural grasslands, while lower altitude sites to the west and northwest of Paraná and also along major rivers valleys, are covered by drier vegetation, the semideciduous forests.

European colonization of Paraná began at the XVI century with few Spanish settlements mainly located near the western state limits, but only towards the XIX century did it’s territory began to be intensely colonized. Large scale deforestation was promoted by exploitation of forests for timber, paving the way for cattle raising and agricultural practices. Today, most of the state of Paraná is dedicated to livestock and plantations, mainly soybean, corn, wheat, and coffee.

Urban areas encompassed in bird sampling are located both inside large cities, with more than 100.000 inhabitants, and smaller urbanized settlements, the smaller town with a population of nearly 4000 people. Within these areas, volunteers visited mainly urban green areas, such as parks, but some residential districts were also sampled. None of the sampled sites was predominantly industrial or commercial.

**S1 Fig.** **Map of the state of Paraná, depicting sampled sites used in the analysis**. Symbols indicate sites embedded in natural (circles), rural (stars) and urban (triangles) landscapes, while phytogeographical categories are shown by symbols with different colors: dense forest (light gray), mixed forest (dark gray) and semideciduous forest (black).

*Bird data*. The Participative Inventory of the Birds of Paraná (IPAVE) is an annual full–week citizen science initiative that began in 2012. At the time, there was broad dissemination of the action through various internet tools in order to engage volunteers to participate in the event. Through social networks and forums specially designed for IPAVE, participants received guidance and instructions for maintaining their records, among which were the distribution of standardized spreadsheets, working protocols, assistance with bird identification from a number of experts who composed the organizing committee and accession of additional information. In the end there were 158 volunteer participants, including birdwatchers, photographers, students and professional ornithologists. Special attention was devoted to the fulfilment of minimum protocols for recording georeferenced information for the localities, as well as the proper identification of the species encountered by participants. All records were analyzed to evaluate their taxonomic and biogeographic validity and, when they elicited doubt, the authors were requested to send additional documentation, such as photos or audio recordings. In the absence of these files, or if they were unreliable, the record was deleted from the list for that site.

Identifications were based exclusively on visual and / or auditory records of the species, eventually associated with photography, videography or audio documentation. In accordance with Brazilian legislation, these methods do not require special approval by faunal authorities or animal ethics committees for volunteers to conduct sampling. Simultaneous data collection was performed between 22 and 30 September 2012, at the beginning of the breeding season, when birds’ detectability is high. Sampling time devoted by volunteers at each site was quantified as hours of observation, independently of how many persons were involved (which span from 1 to 9). Altogether, during IPAVE 2012 134 locations were visited by the participants (erroneously cited as 135 in the original work), including 10 sites that refer to loose records which were discarded from our study. In each site, volunteers covered random walks along trails, roads or sidewalks, recording all birds seen or heard and taking photographs or audio recordings when identification was uncertain. The coordinating team later assisted in bird identification, discarding records that lacked reliability, i.e. based on each volunteer experience, or when plumage or vocal diagnostics were not clear in photos or audio files. To perform our analysis we only considered terrestrial birds, excluding records of waterbird species, namely those in the following families (exceptions are species that occur in grassland and pasture habitats, *sensu* [2]): Anatidae, Podicipedidae, Spheniscidae, Procellariidae, Ciconiidae, Fregatidae, Sulidae, Phalacrocoracidae, Anhingidae, Ardeidae (except *Bubulcus ibis* and *Syrigma sibilatrix*), Threskiornitidae (except *Theristicus caudatus*), Aramidae, Rallidae, Charadriidae (except *Vanellus chilensis*), Haematopodidae, Recurvirostridae, Scolopacidae, Jacanidae, Laridae, Sternidae, Rhyncopidae and Alcedinidae. Nomenclature follows proposals of CBRO [3] (S1 Table).

**S1 Table. Data from the 2012 Participative Inventory of the Birds of Paraná (IPAVE) summarizing bird richness and sampling effort values for each site, along with its human-altered landscape (HAL) and phytogeographical classification. Site codes were obtained from the original work of Straube *et al*. [4] and the effort is represented in hours of sampling. S: species richness.**

| **Site code** | **Municipality** | **Phytogeography** | **HAL** | **S** | **Effort** | **Latitude** | **Longitude** |
| --- | --- | --- | --- | --- | --- | --- | --- |
| GQ-SM^1^ | Guaraqueçaba | Dense | Natural | 173 | 13 | -25.181965 | -48.298772 |
| CG-NH | Campina Grande do Sul | Dense | Natural | 171 | 16 | -25.023333 | -48.619444 |
| MO-BD | Morretes | Dense | Natural | 139 | 50 | -25.602350 | -48.785833 |
| TS-SC | Tijucas do Sul | Mixed | Natural | 135 | 4 | -25.958911 | -49.253436 |
| PG-IT | Ponta Grossa | Mixed | Natural | 133 | 24 | -25.116111 | -49.917778 |
| CR-PA | Cruz Machado | Mixed | Rural | 131 | 10 | -25.886381 | -51.224722 |
| PS-4N | Piraí do Sul | Mixed | Natural | 129 | 13 | -24.363078 | -49.987514 |
| AN-VA | Antonina | Dense | Natural | 124 | 13 | -25.425058 | -48.757903 |
| JA-PC | Jaguariaíva | Mixed | Natural | 124 | 13 | -24.171930 | -49.660736 |
| MS-MS | Mauá da Serra | Mixed | Natural | 124 | 8 | -23.950633 | -51.151603 |
| SJ-SC | São José dos Pinhais | Dense | Natural | 124 | 7 | -25.830000 | -48.933056 |
| LO-FT | Londrina | Semideciduous | Natural | 119 | 4 | -23.475258 | -51.217967 |
| RB-RC | Rio Bonito do Iguaçu | Mixed | Natural | 108 | 5 | -25.428167 | -52.647722 |
| FI-BV | Foz do Iguaçu | Semideciduous | Urban | 106 | 33 | -25.425778 | -54.575000 |
| PP-PS | Pontal do Paraná | Dense | Urban | 105 | 49 | -25.573506 | -48.350239 |
| AN-EG | Antonina | Dense | Natural | 103 | 10 | -25.314636 | -48.700972 |
| LO-FC | Londrina | Semideciduous | Rural | 103 | 4 | -23.501246 | -51.077452 |
| RN-ST | Rio Negro | Mixed | Rural | 102 | 13 | -26.082136 | -49.801133 |
| CL-IT^1^ | Campo Largo | Mixed | Rural | 100 | 9 | -25.421167 | -49.605212 |
| GQ-SU | Guaraqueçaba | Dense | Natural | 99 | 17 | -25.364381 | -48.154692 |
| MO-PC | Morretes | Dense | Rural | 98 | 10 | -25.443661 | -48.875228 |
| MO-PO | Morretes | Dense | Urban | 98 | 36 | -25.490807 | -48.823441 |
| AN-PP | Antonina | Dense | Natural | 96 | 6 | -25.475033 | -48.685412 |
| GU-BS | Guaratuba | Dense | Natural | 91 | 10 | -25.974389 | -48.598369 |
| SE-RF | Sengés | Mixed | Rural | 88 | 8 | -24.119747 | -49.390178 |
| FE-RV | Fenix | Semideciduous | Rural | 86 | 14 | -23.884750 | -51.975072 |
| CU-ZO | Curitiba | Mixed | Urban | 83 | 18 | -25.559329 | -49.230931 |
| QI-SO | Quedas do Iguaçu | Semideciduous | Rural | 83 | 6 | -25.524150 | -53.035031 |
| FE-EP^1^ | Fenix | Semideciduous | Rural | 82 | 7 | -23.893650 | -51.962142 |
| CM-R5^1^ | Campo Mourão | Mixed | Rural | 79 | 9 | -23.995583 | -52.396681 |
| MI-FM | Miraselva | Semideciduous | Rural | 79 | 7 | -22.972236 | -51.419597 |
| AR-RP | Arapoti | Mixed | Rural | 78 | 5 | -24.172433 | -49.916911 |
| BN-BC | Balsa Nova | Mixed | Rural | 78 | 15 | -25.461917 | -49.619556 |
| IB-HF | Ibiporã | Semideciduous | Rural | 78 | 4 | -23.256446 | -51.030845 |
| AN-K9 | Antonina | Dense | Natural | 77 | 50 | -25.318250 | -48.659811 |
| BN-PP | Balsa Nova | Mixed | Natural | 77 | 28 | -25.425833 | -49.718056 |
| FE-VR | Fenix | Semideciduous | Natural | 76 | 7 | -23.917925 | -51.954542 |
| LO-FR | Londrina | Semideciduous | Rural | 70 | 4 | -23.375158 | -51.001361 |
| CU-PB | Curitiba | Mixed | Urban | 67 | 15 | -25.426449 | -49.311217 |
| MO-EA | Morretes | Dense | Rural | 67 | 4 | -25.516426 | -48.840614 |
| CU-BP^1^ | Curitiba | Mixed | Urban | 65 | 7 | -25.416068 | -49.230210 |
| CA-PG^1^ | Cascavel | Mixed | Urban | 62 | 14 | -24.965109 | -53.437081 |
| PL-CA | Palotina | Semideciduous | Urban | 62 | 18 | -24.292819 | -53.842494 |
| CU-UB | Curitiba | Mixed | Urban | 60 | 2 | -25.477639 | -49.221311 |
| MT-CR | Matelândia | Semideciduous | Natural | 60 | 3 | -25.419558 | -53.903278 |
| MA-PI | Maringá | Semideciduous | Urban | 59 | 7 | -23.427036 | -51.931464 |
| NL-RT | Nova Laranjeiras | Mixed | Rural | 59 | 6 | -24.419280 | -52.729390 |
| CM-LA | Campo Mourão | Mixed | Rural | 56 | 4 | -24.110083 | -52.318528 |
| PS-IF | Piraí do Sul | Mixed | Rural | 56 | 6 | -24.522803 | -49.795300 |
| GU-EC | Guaratuba | Dense | Natural | 55 | 2 | -25.857222 | -48.925833 |
| CU-AB | Curitiba | Mixed | Urban | 54 | 4 | -25.379171 | -49.270823 |
| FE-FE | Fenix | Semideciduous | Urban | 54 | 7 | -23.915172 | -51.978906 |
| FI-TR | Foz do Iguaçu | Semideciduous | Natural | 52 | 12 | -25.624408 | -54.474183 |
| GN-NB | Guaraniaçu | Semideciduous | Rural | 52 | 1 | -24.817276 | -52.858786 |
| SJ-PS | São José dos Pinhais | Mixed | Urban | 52 | 6 | -25.512431 | -49.205722 |
| CU-PT | Curitiba | Mixed | Urban | 50 | 8 | -25.402231 | -49.305419 |
| PP-RG | Pontal do Paraná | Dense | Natural | 50 | 12 | -25.672162 | -48.512040 |
| CU-CP | Curitiba | Mixed | Urban | 49 | 6 | -25.450000 | -49.250000 |
| CR-RA | Cruz Machado | Mixed | Rural | 48 | 5 | -26.028139 | -51.335286 |
| CU-CC | Curitiba | Mixed | Urban | 47 | 3 | -25.460103 | -49.324737 |
| CA-PA | Cascavel | Mixed | Rural | 46 | 4 | -24.995000 | -53.290278 |
| DD-ER | Diamante D'Oeste | Semideciduous | Rural | 45 | 7 | -24.936614 | -54.102656 |
| BI-FA | Bituruna | Mixed | Rural | 44 | 6 | -26.068528 | -51.667861 |
| LS-LP | Laranjeiras do Sul | Mixed | Rural | 44 | 2 | -25.335939 | -52.360217 |
| RI-HF | Reserva do Iguaçu | Mixed | Natural | 43 | 5 | -25.792136 | -52.097883 |
| BS-ZR | Bom Sucesso do Sul | Mixed | Rural | 42 | 4 | -26.038764 | -52.888161 |
| TB-VM | Telêmaco Borba | Semideciduous | Rural | 42 | 30 | -24.059016 | -50.708842 |
| MA-CP | Maringá | Semideciduous | Urban | 41 | 5 | -23.402203 | -51.963408 |
| CG-PP | Campina Grande do Sul | Dense | Natural | 40 | 14 | -25.256389 | -48.810278 |
| CG-FP | Campina Grande do Sul | Mixed | Natural | 39 | 5 | -25.221389 | -48.858611 |
| MA-CM | Maringá | Semideciduous | Urban | 39 | 2 | -23.403692 | -51.948819 |
| PL-SC | Palotina | Semideciduous | Rural | 39 | 4 | -24.317950 | -53.912378 |
| PB-PB | Pato Branco | Mixed | Urban | 38 | 5 | -25.568750 | -49.907572 |
| AP-CI | Alto Paraíso | Semideciduous | Rural | 37 | 7 | -23.575459 | -53.901551 |
| CM-SN^1^ | Campo Mourão | Mixed | Urban | 37 | 16 | -24.033806 | -52.396306 |
| PM-CI | Palmas | Mixed | Urban | 37 | 8 | -26.510150 | -51.988461 |
| MA-EZ | Maringá | Semideciduous | Rural | 36 | 3 | -23.397953 | -51.834606 |
| CU-JB | Curitiba | Mixed | Urban | 35 | 4 | -25.442397 | -49.236970 |
| GP-CC | Guarapuava | Mixed | Urban | 35 | 4 | -25.384444 | -51.488333 |
| GP-CE | Guarapuava | Mixed | Rural | 34 | 5 | -25.300556 | -51.445278 |
| NA-SR | Nova Aurora | Semideciduous | Rural | 33 | 6 | -24.525342 | -53.309175 |
| NA-SB | Nova Aurora | Semideciduous | Rural | 32 | 5 | -24.529364 | -53.294775 |
| FI-FI | Foz do Iguaçu | Semideciduous | Urban | 31 | 2 | -25.498722 | -54.565583 |
| CU-BR | Curitiba | Mixed | Urban | 30 | 2 | -25.405653 | -49.280731 |
| MA-Z2 | Maringá | Semideciduous | Urban | 30 | 3 | -23.430725 | -51.937217 |
| SM-SF | Santa Mariana | Semideciduous | Rural | 30 | 4 | -23.165058 | -50.339989 |
| TA-DS | Tamarana | Mixed | Rural | 30 | 8 | -23.739435 | -51.142345 |
| CM-PB^2^ | Campo Mourão | Mixed | Urban | 29 | 2 | -24.055389 | -52.362000 |
| OR-LB^2^ | Ortigueira | Mixed | Natural | 29 | 6 | -24.213256 | -50.690953 |
| PA-LF^2^ | Porto Amazonas | Mixed | Natural | 29 | 2 | -25.574033 | -49.887414 |
| NA-FM^2^ | Nova Aurora | Semideciduous | Natural | 28 | 12 | -24.520947 | -53.179408 |
| IC-PC^2^ | Icaraíma | Semideciduous | Urban | 27 | 2 | -23.365850 | -53.748631 |
| AL-CP^2^ | Altônia | Semideciduous | Rural | 26 | 5 | -23.932778 | -54.019167 |
| LO-MG^2^ | Londrina | Semideciduous | Natural | 26 | 3 | -23.442592 | -51.242661 |
| QB-CO^2^ | Quatro Barras | Dense | Natural | 25 | 4 | -25.335819 | -48.909286 |
| CU-UN^2^ | Curitiba | Mixed | Urban | 24 | 2 | -25.398056 | -49.282500 |
| PP-PL^2^ | Pontal do Paraná | Dense | Urban | 24 | 4 | -25.698136 | -48.468333 |
| PS-FN^2^ | Piraí do Sul | Mixed | Rural | 24 | 4 | -24.572500 | -49.928056 |
| CA-DG^2^ | Cascavel | Mixed | Urban | 23 | 2 | -24.949019 | -53.432228 |
| PA-PA^2^ | Porto Amazonas | Mixed | Urban | 22 | 4 | -25.549953 | -49.889689 |
| MA-CC^2^ | Maringá | Semideciduous | Urban | 21 | 8 | -23.434108 | -51.947167 |
| GP-PL^2^ | Guarapuava | Mixed | Urban | 18 | 7 | -25.398511 | -51.472150 |
| PA-RS^2^ | Porto Amazonas | Mixed | Rural | 18 | 3 | -25.497092 | -49.863039 |
| QB-RJ^2^ | Quatro Barras | Mixed | Natural | 18 | 3 | -25.402331 | -48.984714 |
| FI-JI^2^ | Foz do Iguaçu | Semideciduous | Urban | 17 | 3 | -25.484242 | -54.567844 |
| CU-RM^2^ | Curitiba | Mixed | Urban | 16 | 5 | -25.489869 | -49.260889 |
| CU-SL^2^ | Curitiba | Mixed | Urban | 16 | 2 | -25.400769 | -49.270619 |
| GP-PA^2^ | Guarapuava | Mixed | Urban | 16 | 3 | -25.352856 | -51.466722 |
| AP-CX^2^ | Alto Paraíso | Semideciduous | Rural | 13 | 7 | -23.422500 | -53.830833 |
| CO-RG^2^ | Colombo | Mixed | Urban | 13 | 4 | -25.339825 | -49.156750 |
| UM-BU^2^ | Umuarama | Semideciduous | Urban | 13 | 1 | -23.756406 | -53.309167 |
| PA-LB^2^ | Porto Amazonas | Mixed | Natural | 9 | 3 | -25.568750 | -49.907572 |
| AP-TB^2^ | Alto Paraíso | Semideciduous | Rural | 6 | 1 | -23.376969 | -53.750581 |
| GU-RB^2^ | Guaratuba | Dense | Natural | 1 | 2 | -25.868344 | -48.619783 |

^1^Sites that had multiple and close (< 0.5 km) sampled localities pooled.

^2^Sites with total number of species less than the first quartile of the richness distribution (Q_1_ = 30), excluded from our analysis.

*Landscape categorization*. Each site was classified as part of a natural, rural, or urban setting according to the degree of human-induced alteration of its surroundings. This was determined by considering a circular area with a 5 km radius around the central coordinates of each site, available at [4]. Following subjective visual assessment, each site was assigned to a category when over 80% of the soil cover of the circular area evaluated was composed of native vegetation, agricultural land, or impermeable soil, respectively, for the natural, rural and urban categories. Native vegetation consisted mainly of forested habitat, but some natural grasslands inserted among forest remnants were also considered in this category. Agricultural practices in the state span crop fields and pastures, but also lightly urbanized areas, such as orchards and farmlands in general. Finally, impermeable soil included mainly constructed areas, both from edifications (housings, industries or commerce) and roads.

*Sampling effort bias*. Avifaunal richness recorded at a site is known to be substantially influenced by sampling effort. Considering that all IPAVE participants could allocate their sampling time freely in their selected birding localities, differences among sites could result in variable biases, thus influencing recorded bird composition present at each site. To evaluate this, we have performed a fixed-effect generalized linear model (GLM) model with Poisson distribution [5] to test the effect of HALs and phytogeographical categories, and sampling effort, performed in R [6]. We set the natural areas in dense forest as the intercept, comparing remaining categories with it. All categories showed significant differences in species richness (S2 Table), but the effect of sampling effort were the least important considering the estimates’ coefficient. Also, it is notable that urban areas had the lowest species richness and, as expected, natural areas in dense forest had the highest species richness (S2 Table). .

**S2 Table**. Generalized linear model (GLM) results considering sampling effort (in hours), human-altered landscapes, and phytogeographical categories as predictors of species richness.

|  | Coefficient estimate | Standard error | Z-statistics | *P*-value |
| --- | --- | --- | --- | --- |
| Intercept: Natural sites –Dense forest | 4.484 | 0.035 | 127.450 | < 0.001 |
| Effort (hours) | 0.011 | 0.001 | 8.936 | < 0.001 |
| Semideciduous fores | -0.185 | 0.042 | -4.404 | < 0.001 |
| Mixed forest | -0.072 | 0.037 | -1.934 | 0.053 |
| Rural sites | -0.300 | 0.034 | -8.911 | < 0.001 |
| Urban sites | -0.509 | 0.036 | -14.118 | < 0.001 |

We further tested possible biases caused by differences in sampling effort among sites by performing all analyses with a selected subset of sites. The subset was obtained by analyzing a histogram prepared with sampling effort values and selecting the highest proportion of localities with similar sampling effort. This resulted in 49 sites with sampling effort between 3 and 8 hours. After controlling for sampling effort difference in this manner, the results obtained from analyzing this subset was similar to the pattern observed with our whole set of sites (S3 Table), suggesting that sampling effort differences do not affect the interpretation of our analysis.

**S3 Table**. Average distance to centroid in each group of sites from human-altered landscape (HAL) and phytogeographical categories, as obtained from a multivariate homogeneity of dispersion analysis, computed with a partial dataset (see text). This metric corresponds to our beta diversity value used to assess the degree of homogenization of Atlantic Forest bird assemblages. Statistically significant differences in distance to centroid means are depicted by different letters.

| **Category** | **Group** | **Beta diversity** |  |
| --- | --- | --- | --- |
| **HAL** | Natural | 0.39756 a |  |
|  | Rural | 0.29395 a |  |
|  | Urban | 0.05892 b |  |
| **Phytogeography** | Dense | 0.02296 a |  |
|  | Semideciduous | 0.26553 b |  |
|  | Mixed | 0.22379 b |  |

*Spatial autocorrelation*. We tested whether compositional dissimilarity of avifaunas in each group of categories (HALs and phytogeography) was correlated with geographical distances between sites. The role of pure geographic distance is known to be of great importance in determining composition similarity between two sites, thus this potential bias must be taken into account [7, 8]. To address this issue, we tested the correlation of an Euclidean distance matrix built from geographical coordinate data for each site, with another matrix consisting of avifaunal Raup–Crick dissimilarities in each of the categories with sequential Mantel tests [9], both in Past 3.01 with 9999 permutations [10], and using the function *mantel* in the *vegan* package, with 999 permutations [11] in R software [6]. We found that composition dissimilarity was independent of geographical distance in the natural (r_natural_ = 0.05; p = 0.15) and rural categories (r_rural_ = -0.05; p = 0.9), whereas it was weak but marginally significant in urban settings (r_urban_ = 0.08; p = 0.07). Bird similarity in all categories of phytogeographical domains proved to be independent of the geographic distances between sites (Mantel tests: r_dense_ = -0.151; p = 0.86; r_semideciduous_ = 0.015; p = 0.34; r_mixed_ = 0.008; p = 0.45). Based on these results we assume that spatial autocorrelation was not influential on patterns of bird assemblage compositional dissimilarity in any of the categories analyzed. We further tested spatial autocorrelation using the residuals of the aforementioned GLM to construct a correlogram, which showed that data is not spatially autocorrelated (S2 Fig.).

**S2 Fig. Spatial correlogram of generalized linear model (GLM) residuals.** The correlogram depicts Moran’s I for several distance classes, based on the residuals of a GLM considering species richness as a function of sampling effort , human-altered landscape and phytogeographical categories (see model description in SI).

**S4 Table**. Full indicator bird species list and their indicator values (IndVal) for each of the human-altered landscape (HAL) and phytogeographical categories and calculated p-values. Species are arranged by decreasing IndVal.

| **Species characteristic of...** |  |  |  |  |  |  |
| --- | --- | --- | --- | --- | --- | --- |
| **HAL** | **IndVal** | **p-value** |  | **Phytogeography** | **IndVal** | **p-value** |
| **Natural** |  |  |  | **Dense forest** |  |  |
| *Crypturellus obsoletus* | 0.519 | 0.001 |  | *Tangara cyanocephala* | 0.824 | 0.001 |
| *Cathartes aura* | 0.480 | 0.001 |  | *Turdus flavipes* | 0.765 | 0.001 |
| *Chiroxiphia caudata* | 0.460 | 0.001 |  | *Tangara seledon* | 0.731 | 0.001 |
| *Thalurania glaucopis* | 0.453 | 0.001 |  | *Phaeothlypis rivularis* | 0.672 | 0.001 |
| *Turdus albicollis* | 0.442 | 0.001 |  | *Trogon viridis* | 0.647 | 0.001 |
| *Procnias nudicollis* | 0.441 | 0.001 |  | *Attila rufus* | 0.647 | 0.001 |
| *Sittasomus griseicapillus* | 0.403 | 0.001 |  | *Legatus leucophaius* | 0.647 | 0.001 |
| *Coragyps atratus* | 0.390 | 0.004 |  | *Ramphocelus bresilius* | 0.647 | 0.001 |
| *Rupornis magnirostris* | 0.381 | 0.007 |  | *Thraupis cyanoptera* | 0.647 | 0.001 |
| *Basileuterus culicivorus* | 0.381 | 0.004 |  | *Euphonia pectoralis* | 0.641 | 0.001 |
| *Pionus maximiliani* | 0.369 | 0.001 |  | *Chiroxiphia caudata* | 0.636 | 0.001 |
| *Schiffornis virescens* | 0.367 | 0.001 |  | *Procnias nudicollis* | 0.631 | 0.001 |
| *Tachyphonus coronatus* | 0.357 | 0.001 |  | *Forpus xanthopterygius* | 0.628 | 0.001 |
| *Pyriglena leucoptera* | 0.352 | 0.001 |  | *Euphonia violacea* | 0.628 | 0.001 |
| *Tangara cyanocephala* | 0.345 | 0.001 |  | *Amazilia versicolor* | 0.622 | 0.001 |
| *Cacicus haemorrhous* | 0.344 | 0.02 |  | *Thalurania glaucopis* | 0.618 | 0.001 |
| *Synallaxis spixi* | 0.340 | 0.01 |  | *Brotogeris tirica* | 0.599 | 0.001 |
| *Dacnis cayana* | 0.318 | 0.003 |  | *Celeus flavescens* | 0.556 | 0.001 |
| *Trichothraupis melanops* | 0.312 | 0.002 |  | *Patagioenas plumbea* | 0.544 | 0.001 |
| *Turdus flavipes* | 0.308 | 0.001 |  | *Cantorchilus longirostris* | 0.529 | 0.001 |
| *Phaeothlypis rivularis* | 0.308 | 0.002 |  | *Thraupis palmarum* | 0.525 | 0.001 |
| *Vireo olivaceus* | 0.308 | 0.024 |  | *Vireo olivaceus* | 0.506 | 0.001 |
| *Melanerpes flavifrons* | 0.301 | 0.003 |  | *Synallaxis spixi* | 0.503 | 0.001 |
| *Xiphorhynchus fuscus* | 0.300 | 0.004 |  | *Coereba flaveola* | 0.490 | 0.002 |
| *Phaethornis eurynome* | 0.297 | 0.001 |  | *Myiozetetes similis* | 0.490 | 0.001 |
| *Tangara seledon* | 0.291 | 0.001 |  | *Cyanocorax caeruleus* | 0.486 | 0.001 |
| *Brotogeris tirica* | 0.289 | 0.012 |  | *Turdus albicollis* | 0.476 | 0.001 |
| *Euphonia pectoralis* | 0.285 | 0.002 |  | *Ramphodon naevius* | 0.471 | 0.001 |
| *Pyrrhura frontalis* | 0.283 | 0.019 |  | *Aphantochroa cirrochloris* | 0.471 | 0.001 |
| *Forpus xanthopterygius* | 0.278 | 0.003 |  | *Myrmeciza squamosa* | 0.471 | 0.001 |
| *Celeus flavescens* | 0.278 | 0.003 |  | *Hemithraupis ruficapilla* | 0.471 | 0.001 |
| *Tapera naevia* | 0.278 | 0.008 |  | *Pyriglena leucoptera* | 0.461 | 0.001 |
| *Sirystes sibilator* | 0.275 | 0.001 |  | *Crypturellus obsoletus* | 0.450 | 0.001 |
| *Eleoscytalopus indigoticus* | 0.271 | 0.001 |  | *Todirostrum poliocephalum* | 0.448 | 0.001 |
| *Dryocopus lineatus* | 0.266 | 0.02 |  | *Tachyphonus coronatus* | 0.444 | 0.001 |
| *Odontophorus capueira* | 0.260 | 0.002 |  | *Cathartes aura* | 0.430 | 0.003 |
| *Platyrinchus mystaceus* | 0.259 | 0.004 |  | *Synallaxis ruficapilla* | 0.426 | 0.001 |
| *Euphonia violacea* | 0.255 | 0.007 |  | *Xiphorhynchus fuscus* | 0.422 | 0.001 |
| *Cyanocorax caeruleus* | 0.249 | 0.009 |  | *Picumnus temminckii* | 0.418 | 0.001 |
| *Hemithraupis ruficapilla* | 0.243 | 0.001 |  | *Myrmotherula unicolor* | 0.412 | 0.001 |
| *Elanoides forficatus* | 0.241 | 0.031 |  | *Coragyps atratus* | 0.404 | 0.011 |
| *Patagioenas cayennensis* | 0.240 | 0.011 |  | *Progne chalybea* | 0.394 | 0.002 |
| *Tolmomyias sulphurescens* | 0.237 | 0.007 |  | *Basileuterus culicivorus* | 0.394 | 0.006 |
| *Falco sparverius* | 0.236 | 0.029 |  | *Manacus manacus* | 0.391 | 0.001 |
| *Trogon viridis* | 0.235 | 0.003 |  | *Philydor atricapillus* | 0.379 | 0.001 |
| *Stephanophorus diadematus* | 0.235 | 0.003 |  | *Tyrannus melancholicus* | 0.371 | 0.016 |
| *Attila rufus* | 0.235 | 0.004 |  | *Pygochelidon cyanoleuca* | 0.367 | 0.018 |
| *Tinamus solitarius* | 0.231 | 0.001 |  | *Turdus amaurochalinus* | 0.363 | 0.025 |
| *Phaethornis squalidus* | 0.231 | 0.002 |  | *Saltator similis* | 0.356 | 0.016 |
| *Colonia colonus* | 0.230 | 0.035 |  | *Schiffornis virescens* | 0.354 | 0.001 |
| *Thraupis cyanoptera* | 0.229 | 0.007 |  | *Rupornis magnirostris* | 0.353 | 0.045 |
| *Ramphocelus bresilius* | 0.229 | 0.009 |  | *Tinamus solitarius* | 0.353 | 0.001 |
| *Piculus aurulentus* | 0.224 | 0.005 |  | *Drymophila squamata* | 0.353 | 0.001 |
| *Chamaeza campanisona* | 0.224 | 0.005 |  | *Ilicura militaris* | 0.353 | 0.001 |
| *Pachyramphus castaneus* | 0.224 | 0.005 |  | *Phylloscartes kronei* | 0.353 | 0.001 |
| *Dysithamnus mentalis* | 0.220 | 0.041 |  | *Tachyphonus cristatus* | 0.353 | 0.001 |
| *Patagioenas plumbea* | 0.219 | 0.009 |  | *Tangara peruviana* | 0.353 | 0.001 |
| *Myiopagis caniceps* | 0.219 | 0.012 |  | *Mionectes rufiventris* | 0.351 | 0.001 |
| *Amazilia versicolor* | 0.219 | 0.013 |  | *Cacicus haemorrhous* | 0.347 | 0.024 |
| *Buteo brachyurus* | 0.216 | 0.004 |  | *Dacnis cayana* | 0.325 | 0.004 |
| *Baryphthengus ruficapillus* | 0.212 | 0.014 |  | *Stelgidopteryx ruficollis* | 0.315 | 0.008 |
| *Tityra inquisitor* | 0.206 | 0.009 |  | *Herpsilochmus rufimarginatus* | 0.312 | 0.002 |
| *Mionectes rufiventris* | 0.204 | 0.005 |  | *Geothlypis aequinoctialis* | 0.310 | 0.016 |
| *Lochmias nematura* | 0.194 | 0.012 |  | *Eleoscytalopus indigoticus* | 0.306 | 0.001 |
| *Xenops rutilans* | 0.194 | 0.013 |  | *Carpornis cucullata* | 0.294 | 0.001 |
| *Hylophilus poicilotis* | 0.194 | 0.015 |  | *Ramphocaenus melanurus* | 0.294 | 0.001 |
| *Ramphocaenus melanurus* | 0.192 | 0.007 |  | *Thraupis ornata* | 0.294 | 0.001 |
| *Thraupis ornata* | 0.192 | 0.007 |  | *Sporophila frontalis* | 0.294 | 0.001 |
| *Sporophila frontalis* | 0.192 | 0.007 |  | *Florisuga fusca* | 0.287 | 0.003 |
| *Carpornis cucullata* | 0.192 | 0.008 |  | *Chaetura meridionalis* | 0.272 | 0.013 |
| *Pionopsitta pileata* | 0.190 | 0.023 |  | *Phaethornis squalidus* | 0.272 | 0.002 |
| *Streptoprocne zonaris* | 0.186 | 0.027 |  | *Machetornis rixosa* | 0.260 | 0.035 |
| *Herpsilochmus rufimarginatus* | 0.184 | 0.015 |  | *Cnemotriccus fuscatus* | 0.257 | 0.009 |
| *Legatus leucophaius* | 0.180 | 0.021 |  | *Colonia colonus* | 0.250 | 0.023 |
| *Ramphodon naevius* | 0.179 | 0.011 |  | *Sirystes sibilator* | 0.247 | 0.011 |
| *Myrmeciza squamosa* | 0.179 | 0.013 |  | *Elanoides forficatus* | 0.243 | 0.033 |
| *Chaetura cinereiventris* | 0.179 | 0.02 |  | *Leptopogon amaurocephalus* | 0.243 | 0.033 |
| *Crypturellus tataupa* | 0.177 | 0.044 |  | *Elaenia obscura* | 0.237 | 0.003 |
| *Philydor rufum* | 0.176 | 0.022 |  | *Terenura maculata* | 0.235 | 0.001 |
| *Hypoedaleus guttatus* | 0.176 | 0.03 |  | *Drymophila ferruginea* | 0.235 | 0.001 |
| *Myiornis auricularis* | 0.176 | 0.033 |  | *Formicarius colma* | 0.235 | 0.001 |
| *Geotrygon montana* | 0.173 | 0.018 |  | *Orthogonys chloricterus* | 0.235 | 0.001 |
| *Automolus leucophthalmus* | 0.172 | 0.032 |  | *Conopophaga melanops* | 0.235 | 0.002 |
| *Trogon rufus* | 0.168 | 0.013 |  | *Myiobius barbatus* | 0.235 | 0.002 |
| *Campephilus robustus* | 0.168 | 0.015 |  | *Odontophorus capueira* | 0.234 | 0.006 |
| *Tachyphonus cristatus* | 0.167 | 0.013 |  | *Hypoedaleus guttatus* | 0.234 | 0.007 |
| *Ilicura militaris* | 0.160 | 0.018 |  | *Hylophilus poicilotis* | 0.229 | 0.009 |
| *Phylloscartes kronei* | 0.160 | 0.021 |  | *Chaetura cinereiventris* | 0.229 | 0.002 |
| *Habia rubica* | 0.154 | 0.029 |  | *Fluvicola nengeta* | 0.229 | 0.002 |
| *Pteroglossus bailloni* | 0.154 | 0.007 |  | *Geotrygon montana* | 0.229 | 0.007 |
| *Streptoprocne biscutata* | 0.154 | 0.011 |  | *Amazilia fimbriata* | 0.214 | 0.004 |
| *Orthogonys chloricterus* | 0.154 | 0.015 |  | *Xenops minutus* | 0.214 | 0.004 |
| *Drymophila ferruginea* | 0.154 | 0.017 |  | *Lurocalis semitorquatus* | 0.214 | 0.005 |
| *Conopophaga melanops* | 0.154 | 0.023 |  | *Myiarchus ferox* | 0.207 | 0.019 |
| *Cnemotriccus fuscatus* | 0.150 | 0.04 |  | *Haplospiza unicolor* | 0.188 | 0.011 |
| *Mackenziaena leachii* | 0.148 | 0.021 |  | *Chamaeza campanisona* | 0.183 | 0.028 |
| *Emberizoides herbicola* | 0.148 | 0.021 |  | *Habia rubica* | 0.183 | 0.037 |
| *Lepidocolaptes falcinellus* | 0.143 | 0.048 |  | *Nyctidromus albicollis* | 0.179 | 0.047 |
| *Drymophila malura* | 0.130 | 0.036 |  | *Orchesticus abeillei* | 0.176 | 0.003 |
| *Drymophila rubricollis* | 0.130 | 0.041 |  | *Phylloscartes oustaleti* | 0.176 | 0.004 |
| *Batara cinerea* | 0.130 | 0.049 |  | *Anabazenops fuscus* | 0.176 | 0.005 |
| *Sporophila angolensis* | 0.115 | 0.036 |  | *Conopias trivirgatus* | 0.176 | 0.006 |
| *Saltator fuliginosus* | 0.115 | 0.042 |  | *Malacoptila striata* | 0.176 | 0.007 |
| *Orchesticus abeillei* | 0.115 | 0.042 |  | *Ortalis guttata* | 0.176 | 0.008 |
| *Knipolegus nigerrimus* | 0.115 | 0.044 |  | *Hirundo rustica* | 0.176 | 0.008 |
| *Malacoptila striata* | 0.115 | 0.046 |  | *Pseudastur polionotus* | 0.176 | 0.009 |
| *Grallaria varia* | 0.115 | 0.046 |  | *Grallaria varia* | 0.176 | 0.009 |
| *Conopias trivirgatus* | 0.115 | 0.047 |  | *Crypturellus noctivagus* | 0.176 | 0.01 |
| *Elaenia mesoleuca* | 0.115 | 0.049 |  | *Ramphastos vitellinus* | 0.176 | 0.01 |
| **Rural** |  |  |  | *Dysithamnus stictothorax* | 0.176 | 0.01 |
| *Trogon surrucura* | 0.287 | 0.026 |  | *Myrmotherula gularis* | 0.176 | 0.012 |
| **Urban** |  |  |  | *Tiaris fuliginosus* | 0.155 | 0.011 |
| *Passer domesticus* | 0.456 | 0.001 |  | *Dendrocincla turdina* | 0.147 | 0.022 |
| *Furnarius rufus* | 0.438 | 0.001 |  | *Hydropsalis torquata* | 0.139 | 0.018 |
| *Pygochelidon cyanoleuca* | 0.400 | 0.002 |  | *Piculus flavigula* | 0.118 | 0.036 |
| *Columba livia* | 0.394 | 0.001 |  | *Stymphalornis acutirostris* | 0.118 | 0.036 |
| *Eupetomena macroura* | 0.387 | 0.001 |  | *Agelasticus cyanopus* | 0.118 | 0.036 |
| *Zenaida auriculata* | 0.381 | 0.002 |  | *Phylloscartes sylviolus* | 0.118 | 0.041 |
| *Molothrus bonariensis* | 0.357 | 0.035 |  | *Amazona brasiliensis* | 0.118 | 0.042 |
| *Colaptes campestris* | 0.349 | 0.021 |  | **Semideciduous forest** |  |  |
| *Estrilda astrild* | 0.229 | 0.007 |  | *Turdus leucomelas* | 0.556 | 0.001 |
| *Satrapa icterophrys* | 0.219 | 0.012 |  | *Euphonia chlorotica* | 0.504 | 0.001 |
| *Amazona aestiva* | 0.173 | 0.013 |  | *Aratinga leucophthalma* | 0.492 | 0.001 |
| *Elaenia spectabilis* | 0.133 | 0.045 |  | *Zenaida auriculata* | 0.461 | 0.002 |
|  |  |  |  | *Thamnophilus doliatus* | 0.421 | 0.001 |
|  |  |  |  | *Hylocharis chrysura* | 0.367 | 0.001 |
|  |  |  |  | *Columbina picui* | 0.355 | 0.003 |
|  |  |  |  | *Crotophaga ani* | 0.343 | 0.023 |
|  |  |  |  | *Synallaxis frontalis* | 0.332 | 0.001 |
|  |  |  |  | *Todirostrum cinereum* | 0.295 | 0.002 |
|  |  |  |  | *Ictinia plumbea* | 0.288 | 0.007 |
|  |  |  |  | *Chlorostilbon lucidus* | 0.284 | 0.034 |
|  |  |  |  | *Empidonomus varius* | 0.252 | 0.034 |
|  |  |  |  | *Arremon flavirostris* | 0.250 | 0.003 |
|  |  |  |  | *Corythopis delalandi* | 0.250 | 0.005 |
|  |  |  |  | *Conirostrum speciosum* | 0.236 | 0.021 |
|  |  |  |  | *Cissopis leverianus* | 0.228 | 0.027 |
|  |  |  |  | *Myiopagis viridicata* | 0.214 | 0.002 |
|  |  |  |  | *Coryphospingus cucullatus* | 0.190 | 0.042 |
|  |  |  |  | *Elaenia spectabilis* | 0.175 | 0.014 |
|  |  |  |  | *Picumnus cirratus* | 0.175 | 0.016 |
|  |  |  |  | *Icterus pyrrhopterus* | 0.161 | 0.033 |
|  |  |  |  | *Capsiempis flaveola* | 0.143 | 0.026 |
|  |  |  |  | *Pipra fasciicauda* | 0.143 | 0.027 |
|  |  |  |  | *Amazilia lactea* | 0.143 | 0.029 |
|  |  |  |  | *Sporophila collaris* | 0.107 | 0.031 |
|  |  |  |  | *Nemosia pileata* | 0.107 | 0.033 |
|  |  |  |  | *Glaucidium brasilianum* | 0.107 | 0.038 |
|  |  |  |  | *Brotogeris chiriri* | 0.107 | 0.04 |
|  |  |  |  | *Basileuterus flaveolus* | 0.107 | 0.043 |
|  |  |  |  | **Mixed forest** |  |  |
|  |  |  |  | *Theristicus caudatus* | 0.579 | 0.001 |
|  |  |  |  | *Sporagra magellanica* | 0.486 | 0.001 |
|  |  |  |  | *Leucochloris albicollis* | 0.443 | 0.001 |
|  |  |  |  | *Basileuterus leucoblepharus* | 0.440 | 0.001 |
|  |  |  |  | *Colaptes campestris* | 0.436 | 0.001 |
|  |  |  |  | *Leptasthenura setaria* | 0.429 | 0.001 |
|  |  |  |  | *Zonotrichia capensis* | 0.404 | 0.004 |
|  |  |  |  | *Turdus rufiventris* | 0.385 | 0.022 |
|  |  |  |  | *Molothrus bonariensis* | 0.343 | 0.035 |
|  |  |  |  | *Ramphastos dicolorus* | 0.311 | 0.018 |
|  |  |  |  | *Poospiza cabanisi* | 0.310 | 0.003 |
|  |  |  |  | *Thraupis bonariensis* | 0.298 | 0.006 |
|  |  |  |  | *Veniliornis spilogaster* | 0.296 | 0.024 |
|  |  |  |  | *Serpophaga subcristata* | 0.294 | 0.012 |
|  |  |  |  | *Cranioleuca obsoleta* | 0.281 | 0.015 |
|  |  |  |  | *Rhynchotus rufescens* | 0.251 | 0.011 |
|  |  |  |  | *Cacicus chrysopterus* | 0.215 | 0.021 |
|  |  |  |  | *Synallaxis cinerascens* | 0.207 | 0.014 |
|  |  |  |  | *Thamnophilus ruficapillus* | 0.196 | 0.046 |
|  |  |  |  | *Tangara preciosa* | 0.192 | 0.035 |
|  |  |  |  | *Colibri serrirostris* | 0.184 | 0.026 |
|  |  |  |  | *Xolmis cinereus* | 0.160 | 0.034 |

# References

[1] Maack, R. Geografia física do estado do Paraná. 2^nd^ Edition. Rio de Janeiro: José Olympio; 1981.

[2] Parker III TA, Stotz DF, Fitzpatrick JW. Ecological and distribution databases. In: Stotz DF, Fitzpatrick JW, Parker III TA, Moskovits DK, editors. Neotropical birds: ecology and conservation. Chicago: University of Chicago Press; 1996. pp. 131-436.

[3] CBRO (Comitê Brasileiro de Registros Ornitológicos) Listas das aves do Brasil. 11^nd^ Edition. 2014. Available at: http://www.cbro.org.br.

[4] Straube FC, Vallejos MAV, Deconto LR, Urben-Filho A. IPAVE-2012: Inventário Participativo das Aves do Paraná. Curitiba: Hori Consultoria Ambiental; 2013.

[5] Dobson AJ. An introduction to Generalized Linear Models. 2^nd^ Edition. London: Chapman & Hall; 1990.

[6] R Core Development Team. R: a language and environment for statistical computing. Vienna: R Foundation for Statistical Computing; 2012.

[7] McKinney ML. Urbanization as a major cause of biotic homogenization. Biol Conserv. 2006; 127: 247-260.

[8] Kühn I. Incorporating spatial autocorrelation may invert observed patterns. Divers Distr. 2007; 13: 66-69.

[9] Mantel N. The detection of disease clustering and a generalized regression approach. Cancer Res. 1967; 27: 209-220.

[10] Hammer Ø, Harper DAT, Ryan PD. PAST: Paleontological Statistics Software Package for education and data analysis. Palaeontol Electronica. 2001; 4: 1-9.

[11] Oksanen J, Kindt R, O’Hara RB. vegan: community ecology package. R version 1.6-9; 2013.
